# Supplementary material for: One-step automated bioprinting-based method for cumulus-oocyte complex microencapsulation for 3D in vitro maturation
Source: PLoS One. 2020 Sep 11;15(9):e0238812. doi: 10.1371/journal.pone.0238812 (PMC7485809; doi:10.1371/journal.pone.0238812)
Supplement: S1 File — (DOCX) [file pone.0238812.s001.docx]

**S1 file. Effects of 3D IVM culture in alginate engineered microbeads on the maturation rate of adult sheep oocytes.**

| **IVM conditions** | **Alginate concentration**  **(%)** | **n. of analyzed**  **COCs** | **Nuclear chromatin configurations**  **n. (%)** | | | | |
| --- | --- | --- | --- | --- | --- | --- | --- |
|  |  |  | **GV** | **MI to TI** | **MII** | **Abnormal** | **Activated** |
| **2D** | 0 | 153 | 26  (16.99) | 21  (13.73) | 87  (56.86) ^a^ | 19  (12.42) ^a^ | 0  (0) ^a^ |
| **3D** | 1 | 196 | 29  (14.80) | 24 (12.24) | 133 (67.86) ^b,x^ | 10  (5.10) ^b^ | 0  (0) ^a^ |
| **3D** | 2 | 57 | 12  (21.05) | 13 (22.81) | 23  (40.35) ^b,y^ | 5  (8.77) | 4  (7.02) ^c^ |

Table legend: GV= Germinal vesicle; M=metaphase; T=telophase.

Chi square test: within each column, different superscripts indicate statistically significant differences:

- comparison 2D vs 3D 1% alginate: a,b= P<0.05;

- comparison 2D vs 3D 2% alginate: a,b=P<0.05; a,c= P<0.001;

- comparison 3D 1% alginate vs 3D 2% alginate: a,c= P<0.001; x,y= P<0.001.

**Assessment of nuclear maturation rate of oocytes cultured in the 3D IVM system in 2% alginate microbeads**

As further microbead characterization, the maturation rates of oocytes cultured in 2% alginate microbeads were analyzed in 3 replicates. The MII rate in 2% microbeads (23/57; 40%) was significantly lower than those in 1% microbeads (133/196, 68%; P<0.001) and in 2D controls (87/153, 57%; P<0.01). Moreover, in 2% alginate, the percentage of activated oocytes was significantly higher (7% vs 0%; P<0.001).
