# Supplementary material for: One-step automated bioprinting-based method for cumulus-oocyte complex microencapsulation for 3D in vitro maturation
Source: PLoS One. 2020 Sep 11;15(9):e0238812. doi: 10.1371/journal.pone.0238812 (PMC7485809; doi:10.1371/journal.pone.0238812)
Supplement: Attachment — Submitted filename: Response to Reviewers.docx [file pone.0238812.s003.docx]

PONE-D-20-09540R1
One-step automated bioprinting-based method for cumulus-oocyte complex microencapsulation  for 3D in vitro maturation
PLOS ONE

Dear Dr. Mastrorocco,

Thank you for submitting your manuscript to PLOS ONE. After careful consideration, we feel that it has merit but does not fully meet PLOS ONE’s publication criteria as it currently stands. Therefore, we invite you to submit a revised version of the manuscript that addresses the points raised during the review process.

==============================
Please authors, carefully consider each of the questions raised by reviewers # 1 and # 2.
==============================

Please submit your revised manuscript by Aug 16 2020 11:59PM. If you will need more time than this to complete your revisions, please reply to this message or contact the journal office at plosone@plos.org. When you're ready to submit your revision, log on to https://www.editorialmanager.com/pone/ and select the 'Submissions Needing Revision' folder to locate your manuscript file.

Please include the following items when submitting your revised manuscript:

- A rebuttal letter that responds to each point raised by the academic editor and reviewer(s). You should upload this letter as a separate file labeled 'Response to Reviewers'.
- A marked-up copy of your manuscript that highlights changes made to the original version. You should upload this as a separate file labeled 'Revised Manuscript with Track Changes'.
- An unmarked version of your revised paper without tracked changes. You should upload this as a separate file labeled 'Manuscript'.

If you would like to make changes to your financial disclosure, please include your updated statement in your cover letter. Guidelines for resubmitting your figure files are available below the reviewer comments at the end of this letter.

If applicable, we recommend that you deposit your laboratory protocols in protocols.io to enhance the reproducibility of your results. Protocols.io assigns your protocol its own identifier (DOI) so that it can be cited independently in the future. For instructions see: <http://journals.plos.org/plosone/s/submission-guidelines#loc-laboratory-protocols>

We look forward to receiving your revised manuscript.

Kind regards,

Marcelo Fábio Gouveia Nogueira, Associate Professor, Ph. D.
Academic Editor
PLOS ONE


[Note: HTML markup is below. Please do not edit.]

Reviewers' comments:

Reviewer's Responses to Questions

**Comments to the Author**

1. If the authors have adequately addressed your comments raised in a previous round of review and you feel that this manuscript is now acceptable for publication, you may indicate that here to bypass the “Comments to the Author” section, enter your conflict of interest statement in the “Confidential to Editor” section, and submit your "Accept" recommendation.

Reviewer #1: All comments have been addressed

Reviewer #2: All comments have been addressed

Reviewer #3: All comments have been addressed

2. Is the manuscript technically sound, and do the data support the conclusions?

The manuscript must describe a technically sound piece of scientific research with data that supports the conclusions. Experiments must have been conducted rigorously, with appropriate controls, replication, and sample sizes. The conclusions must be drawn appropriately based on the data presented.

Reviewer #1: Yes

Reviewer #2: Yes

Reviewer #3: Yes

3. Has the statistical analysis been performed appropriately and rigorously?

Reviewer #1: I Don't Know

Reviewer #2: Yes

Reviewer #3: Yes

4. Have the authors made all data underlying the findings in their manuscript fully available?

The [PLOS Data policy](http://www.plosone.org/static/policies.action#sharing) requires authors to make all data underlying the findings described in their manuscript fully available without restriction, with rare exception (please refer to the Data Availability Statement in the manuscript PDF file). The data should be provided as part of the manuscript or its supporting information, or deposited to a public repository. For example, in addition to summary statistics, the data points behind means, medians and variance measures should be available. If there are restrictions on publicly sharing data—e.g. participant privacy or use of data from a third party—those must be specified.

Reviewer #1: Yes

Reviewer #2: (No Response)

Reviewer #3: Yes

5. Is the manuscript presented in an intelligible fashion and written in standard English?

PLOS ONE does not copyedit accepted manuscripts, so the language in submitted articles must be clear, correct, and unambiguous. Any typographical or grammatical errors should be corrected at revision, so please note any specific errors here.

Reviewer #1: Yes

Reviewer #2: Yes

Reviewer #3: Yes

6. Review Comments to the Author

Please use the space provided to explain your answers to the questions above. You may also include additional comments for the author, including concerns about dual publication, research ethics, or publication ethics. (Please upload your review as an attachment if it exceeds 20,000 characters)

Reviewer #1: The article titled “One-step automated bioprinting-based method for cumulus-oocyte complex microencapsulation for 3D in vitro maturation” has been substantially improved after the author’s modification. The text is more reader friendly, the aim of the manuscript is clearer, and some information, previously missing, has been added in the text. I am satisfied with the answers addressed to my earlier comments.

In this new submission, the author’s included new analysis to assess the bioenergetic/oxidative status of the oocytes aimed to make cleared the effect of the 3D IVM system in the mitochondria activity, intracellular ROS levels, and mitochondria/ROS colocalization.
Theses analyses corroborates to the data presented earlier, however the discussion of these results need to be improved. The lines 484-486 of the discussion session need reference. And the effect of higher mitochondria activity and ROS levels in the oocyte from 3D-IVM system need to be discussed.

We thank the reviewer for giving us the opportunity to improve the quality of our manuscript. The discussion on results on the effects of 3D IVM on mitochondria activity and ROS levels has been improved, as requested. References for lines 484-486 have been provided. Possible explanations of higher mitochondria activity and ROS levels found in matured oocytes from the 3D-IVM system have been provided.

Specific comments:

Line 37: Change “that” by “than”.

The word has been corrected.

Line 251-252: The definition of spontaneous parthenogenetic activation should be reviewed. Moreover, this classification of activated oocytes was not presented in the results.

Oocyte spontaneous parthenogenetic activation is a post-Metaphase II condition in which chromatin de-condensation and pronuclear formation take place in the absence of sperm penetration and fertilization. This event is related to the decline in the activity of the two major kinases that regulate meiotic cycle (MPF and MAPK; Ariu et al., 2014). In physiological conditions, MPF and MAPK decline occur in association with fertilization in order to allow male (sperm) and female (oocyte Metaphase II) chromatin de-condensation to obtain the two pronuclei. In experimental conditions, parthenogenetic activation is used to test oocyte pronuclear formation ability, thus its developmental competence (Schoevers et al., 2016) and it is frequently used in sheep oocytes and other animal models. In most of dated and recent references, parthenogenetically activated oocytes are defined as those oocytes showing one pronucleus (Ledda et al., 1997; Sun et al., 2002; Jiang et al., 2014), but the first polar body is extruded (See Figure 3D in Ariu et al., 2014). For these reasons, we think that our definition in the Methods section section is correct. However, upon reviewer suggestion, we changed the sentence for clarity and provided a supporting references (Ariu et al., 2014)

The classification of activated oocytes was presented in Supplementary file 1 because oocytes with this chromatin configuration were observed only after IVM in 2% alginate microbeads. A possible explanation for increased rates of activated oocytes in 2% vs 1% or vs control bidimensional IVM could be related to the fact that COCs cultured in microbeads with too high alginate concentration, impeding cumulus expansion, may have undergone dysmetabolic conditions, thus their MPF/MAPK levels could have been reduced, with consequent MII chromatin de-condensation and pronucleus formation.

Lines 371-374: the information in these lines is conflicting. First it says there was a significant difference between 3D and 2D groups in relation to oocyte abnormal chromatin configuration. Followed by a sentence that says: no effect of different systems was noticed on the incidence of specific abnormal chromatin configurations. In figure 5, it shows a difference between 3D and 2D groups in relation to oocyte abnormal chromatin configuration. Please, confirm the information in these lines.

Certainly the reviewer intended to refer to table 5. We confirm the information that the 3D system reduced the incidence of oocyte chromosomal abnormalities. The conflicting sentence has been eliminated, as suggested.

Lines 404-405: Remove the sentence “Oocytes matured in 3D IVM showed significantly increased mitochondrial activity and intracellular ROS levels.” This is not information for a legend.

This sentence has been removed, as requested.

Lines 413-414: Remove the sentence “Increased MitoTracker (C1 versus C2) and DCF fluorescent intensity (D1 versus D2) are visible in 3D IVM oocytes.” This is not information for a legend.

This sentence has been removed, as requested.

Line 416: I would consider changing “improves oocyte molecular maturation” by “modulates oocyte gene expression”. The term “molecular maturation” refers to many other processes and transcripts that were not investigated in this study.

This sentence has been modified, as suggested.

Line 427: Please, consider to change the expression “tended to decrease” by “was similar between groups”. The p-values informed are not consistent with a classification as tendency.

This sentence has been modified, as suggested.

Line 511: Remove the words “reduction (although not significant) of”

The suggested worlds have been removed.

Lines 511-514: I do not understand the comparison between the data from the present study with the GDF-9 quantification analysis by Morselli et al. [34].

In our opinion, our results on down-regulation of expression of genes coding for SCMC complex in 3D IVM are in agreement with those on down-regulated expression of GDF-9 observed in canine after 3D IVM (Morselli et al. [34]). Indeed, similarly to SCMC, GDF-9 is involved in oocyte developmental competence and its expression is reported to decrease during in vivo and in vitro oocyte maturation (De Los Reyes et al., Theriogenology 2013).

Reviewer #2: All the questions raised were appropriately answered by the authors and the changes were incorporated into the text, making it suitable for publication. The only observation I still make is the micrograph representing the chromosomal configuration of the 2D group in Figure 3, as apparently the oocyte has not been fully denuded and the chromatin nof the cumulus cells can hinder the visualization of the oocyte chromatin, leading to errors in the interpretation of the nuclear configuration of the oocyte. oocyte. Therefore, this image should be replaced by one of better representation.

We thank the reviewer for giving us the opportunity to improve the quality of our manuscript. As requested, in Figure 3, the micrograph representing the chromosomal configuration of an oocyte cultured in the 2D system has been replaced with a better one without cumulus cells in order to improve chromatin visualization.

Reviewer #3: The authors have included new experiments and data, that together with corrections made to the manuscript, have improved the work. Questions raised previously were appropriately addressed.

We thank reviewer 3 for these positive comments.

7. PLOS authors have the option to publish the peer review history of their article ([what does this mean?](https://journals.plos.org/plosone/s/editorial-and-peer-review-process#loc-peer-review-history)). If published, this will include your full peer review and any attached files.


If you choose “no”, your identity will remain anonymous but your review may still be made public.


**Do you want your identity to be public for this peer review?** For information about this choice, including consent withdrawal, please see our [Privacy Policy](https://www.plos.org/privacy-policy).

Reviewer #1: No

Reviewer #2: No

Reviewer #3: No

[NOTE: If reviewer comments were submitted as an attachment file, they will be attached to this email and accessible via the submission site. Please log into your account, locate the manuscript record, and check for the action link "View Attachments". If this link does not appear, there are no attachment files.]

While revising your submission, please upload your figure files to the Preflight Analysis and Conversion Engine (PACE) digital diagnostic tool, <https://pacev2.apexcovantage.com/>. PACE helps ensure that figures meet PLOS requirements. To use PACE, you must first register as a user. Registration is free. Then, login and navigate to the UPLOAD tab, where you will find detailed instructions on how to use the tool. If you encounter any issues or have any questions when using PACE, please email PLOS at [figures@plos.org](mailto:figures@plos.org). Please note that Supporting Information files do not need this step.

*In compliance with data protection regulations, you may request that we remove your personal registration details at any time.* [*(Remove my information/details)*](https://www.editorialmanager.com/pone/login.asp?a=r)*. Please contact the publication office if you have any questions.*
